# Embodied Communication, Sensed Atmospheres, Joint Situations: Human–Horse Encounters from a Neophenomenological Perspective

**DOI:** 10.3390/ani14121720

**Published:** 2024-06-07

**Authors:** Robert Pütz

**Affiliations:** Goethe University Frankfurt, 60626 Frankfurt am Main, Germany; puetz@uni-frankfurt.de

**Keywords:** animals and society, new phenomenology, embodied communication, human–animal studies, animal geography, horse, horseback riding

## Abstract

**Simple Summary:**

How can human–animal studies benefit from philosophy, especially the new phenomenology, which was founded by Hermann Schmitz? This article discuss that question, using the example of human–horse relationships. Based on the results of qualitative social research, three basic concepts of new phenomenology are empirically examined and transferred to human–horse encounters: embodied communication, atmosphere, and joint situation.

**Abstract:**

This article shows that the German philosopher Hermann Schmitz’s new phenomenology can make a valuable contribution to human–animal studies. The three concepts suitable for this purpose are, first, Schmitz’s concept of embodied communication, which can be applied to trans-species encounters; second, his understanding of atmospheres, which are always co-communicated in trans-species encounters; and, third, his conception of situation, which can help with analyzing the relationship of society to animals. My contribution applies these three basic elements of new phenomenology—embodied communication, atmosphere, and situation—to the analysis of the encounters between humans and horses. This paper demonstrates that embodied communication in particular is not only a worthwhile object of research but can also serve as a mode of producing scientific insight.

## 1. Introduction

Hermann Schmitz (1928–2021) was a German philosopher. With his ten-volume major work, System of Philosophy, he founded and further elaborated “new phenomenology”. A synopsis of his school of thought was published in English in 2019 [1]. Recently, various authors have labeled Hermann Schmitz’s new phenomenology as inherently transhuman [2] and have further developed it towards a transhuman neophenomenological sociology [3]. This is the point of departure of my contribution, which advocates for recognizing and utilizing the value that it has for human–animal studies in areas where it has not yet found reception. 

In short, I see three starting points for this endeavor. First, as opposed to many other phenomenologies, Schmitz does not move human consciousness to the center of his theory but rather “affective involvement”. Unlike consciousness [4], affective involvement is an inherent characteristic of both humans and sentient animals, which makes it possible in principle to apply his conception to trans-species encounters. Second, sentient beings, whether human or nonhuman, are, according to Schmitz, always in contact with their environment via communication. This he calls “embodied communication” [4] or, in some works, “felt-bodily communication” [1]. Such communication can also encompass things, half-things (like the wind), and atmospheres, which are relevant in communicative situations—whether these situations involve humans or sentient animals. Third, his theory provides an elaborate conception of situations in which embodied communication of any kind is always embedded. In his conception, situations combine the micro and macro levels because superordinate structures (e.g., in the form of “programs”) always take effect in these situations. 

My contribution begins with the micro perspective and focuses on situations of encounters between humans and horses. It does so by addressing a very specific one: horseback riding. The specifics of the relationship between humans and horses mentioned above have rendered it a frequent object of scientific analyses in different fields of human–animal studies: mutually becoming with [5,6,7,8,9], animal work [10,11], wild/feral horses [12,13,14,15], animal welfare [16,17,18], to mention only a few important topics. Horseback riding is a suitable empirical example because it can be viewed at the micro level as a very intense and complex form of embodied trans-species communication, while at the macro level, it is embedded in the larger contexts of the relationships between humans and animals. These involve, for in-stance, the societal relationship to animals in consumer society (e.g., horses and horse breeds as status symbols), the commodification of living beings (e.g., horse breeding and trade), or animal-related services (e.g., horse shows) but also the presence of powerful discourses (e.g., the symbolic meaning of horses, the traditions of court rid-ing schools, and teachings on riding). The focus of this contribution, however, is on embodied communication as the core concept.

The empirical examples that this contribution draws on are from my own ethnographic empirical work that took horse–human encounters as its entry point to address more fundamental questions concerning nature conservation, the commodification of nature, and animal work [13,19,20,21]. In addition, I conducted narrative interviews with a horseback rider and horse owner with whom I am very well acquainted to generate narrations about encounters with horses, which can be considered, in the Schmitzian vein, from the angle of affective involvement, embodied communication, and the significance of atmospheres and situations. These interviews are distinct in various ways from the usual fieldwork in qualitative social research:

The interviewer and interviewee have known each other for many years, so that there was a trusting relationship from the beginning, which allowed the researcher to address issues in ways that would not have been possible with an unfamiliar person.

The interviewer and interviewee share a great passion for horses, and both are engaged in horseback riding so that speaking about their experiences with horses and sharing their emotions and affective involvement in these situations was not unusual (which it would have been in interviews otherwise). This prompted statements from the interviewee that would probably have not been made when engaging with an interviewer who is not a horseback rider.

Per her request, the interviewee was involved in the further processing of her texts. She received the interview transcript for revision and was given the opportunity to condense the interview passages on her own. In this respect, too, the interview material is textually different from the texts that interviewing usually generates.

In addition, I attempted to take a neophenomenologically informed view to the representations of the training of horses and horseback riding in handbooks 

## 2. Horseback Riding as Embodied Communication

### 2.1. Mutual Learning to Be Affected by Riding Aids

At the heart of Schmitz’s conception of a new phenomenology is the concept of “embodied communication” that links both humans and animals with the world. Schmitz distinguishes between one-sided and mutual encorporation ([4], p. 16), which do not occur exclusively between living beings. One-sided encorporation can be found in all situations of fascination or suggestion that are not inflicted by humans (objects, places, landscapes). Mutual encorporation occurs when the interacting participants affect each other and thus their respective actions ([3], p. 189). In terms of their capacity for mutual encorporation, animals are no different from humans in principle. Mutual embodied communication can thus be understood as the basic form of trans-species communication.

Horseback riding stands out among all forms of mutual embodied communication between humans and animals because, more than any other, it represents a complex kind of embodied communication, and people have been engaging with it for millennia. The entire body of horseback riding literature since Xenophon ultimately deals with nothing other than the practice of embodied communication between humans and horses; has for millennia aimed to understand, learn, and teach it down to the most minute details; and—above all—to permanently refine it both among humans and horses.

To conceptualize horseback riding, Schmitz’s distinction between antagonistic und solidary mutual encorporation is useful: encorporation is antagonistic when the relationship between the participants is asymmetric and one of the two ‘calls the shots’; that is, one controls the movements of the collective body that is the product of embodied communication. This can involve a continuous shift in the dominant role between the participants (as in a conversation) ([22], p. 39ff). Solidary encorporation, by contrast, describes a symmetric relationship in which none of the partners adopts a dominant role. 

(In this contribution I concern myself exclusively with modes of riding that, in the broadest sense, fall under the category of equestrian art or subtle horsemanship, that is, that the main objective is to continuously refine the communication with the horse, in particular via the riding aids—like when an artist seeks to refine their modes of expression. Yet, the world of riding knows a wide range of different modes of riding that are geared toward different objectives (e.g., winning contests, breeding horses for profit, delighting a circus audience) that often dominate how horses are dealt with and in which subtle riding aids play only a subordinate role).

Drawing on my ethnographic observations and interviews, I—at this point very generally—conclude that most horsemanship seeks to achieve mutual solidary encorporation, whereas the practice of riding, however, is characterized by the permanent alternation between antagonistic and solidary encorporation, while antagonistic encorporation dominates most of the time (This is meant as a value-free characterization. Of course, antagonistic encorporation can take on a violent form in riding and frequently does (see below)). Ray Hunt, one of the role models of what has been termed natural horsemanship, describes how he seeks to train horses in accordance with the ideal of a kind of horseback riding in which the horse and human unite to become a collective body:

“I try to visualize my body and the horse’s body as one. Since my feet do not touch the ground I think of his feet and legs as being mine. […] When the horse moves and you move with him, your idea and his idea become one. He isn’t dragging you and you aren’t pushing him along. It is feel, timing, and balance” ([23], p. 13).

This statement illustrates two things: First, drawing on Schmitz, we can describe this embodied synchronization as representing mutual encorporation in the solidary form. The riding human feels the horse’s legs as if they were his own. The human tries to synchronize his movements with the horse’s, moves “with him”. This joint movement is characterized by an absence of rough riding aids such as pulling, pushing, and hard whipping. In the practical instruction often encountered at riding schools, we see an emphasis on the material bodies of the rider and horse (e.g., “straighten your back”, “use more left leg” for the human, and “take the head higher” or “more flexion” for horses); the quote emphasizes aspects of the felt body, indicating, for example, that the synchronization of movements does not pertain purely to the material bodies alone but extends also to the synchronization of perceptions and ideas.

In referring to feel, timing, and balance, Hunt denotes three foundations of horsemanship. Frequently labeled differently, they have formed the basis of all teachings on riding for centuries. When feel, timing, or balance are no longer present (mostly, but not always, as the result of an intervention by the rider), according to Schmitz’s conception, solidary turns into antagonistic embodied communication. This is because the rider using riding aids of any kind actively influences the horse’s movements. In line with Schmitz, we can call such riding aids “suggestions of motion” ([4], p. 18), which he considers an essential medium of encorporation. Such suggestions of motion involve not only bodily movements such as pushing or pulling but also rhythmic phenomena such as cadence, impulsion, or, as mentioned in the interview, timing. What this refers to is the temporal concurrence of motion and motional stimulus, which—following Schmitz—enables the partners to act in unison with no notable reaction time in the first place [22]. In this context, feel, timing, and balance often interact—they must be sensed, acquired, and refined in the sense of *learning to be affected*. For instance, an inexperienced rider who fails to properly feel the horse’s movements (perhaps because of concentrating on not falling off instead of adapting to them) might employ a riding aid indicating to the horse “place your right front leg outward” at a moment when the horse cannot do so physically simply because of having its weight on that leg. Just like a goalie can be ‘caught on the wrong foot’, thus leading the person to almost freeze in their movement, or a dancer can provide the proper stimulus to their partner but at the wrong time, thus tripping them up, incorrect timing (as a result of not properly feeling the other) inevitably throws the horse off balance.

Ultimately, all teachings on riding strive for the ideal but rarely achieved end result of a form of solidary encorporation that requires no further riding aids in principle but relies on a shared understanding and joint performance of a sequence of movement. In this context, a riding instructor employed the figure of the centaur in Greek mythology:

“We call these centauric moments, the complete union with the horse. Physically and mentally. These are the moments that our horsemanship strives for.” (Horse trainer, 2020).

In their most basic form, riding aids are stimuli to move or stop, to change pace or gaits (walk, trot, canter), or to change directions. Advanced horsemanship increasingly utilizes aids to prompt the horse to make micro movements. For instance, it may be supposed to shift its weight to its haunches to collect itself, as this makes the horse’s entire body more responsive, enabling more fine-tuned communication, while it is at the same time beneficial to the horse’s health because it can more easily bear the rider’s weight. At the same time, the rider has to anticipate what the horse is offering in order to be synchronized.

A connecting point for developing a neophenomenological perspective of riding aids is Schmitz’s understanding of the “vital drive”, which is “formed by the intertwined tendencies of contraction and expansion” ([1], p. 65). When we apply this concept to embodied communication between human and horse, this means that the two are united by a shared vital drive through expansion/tension or contraction/swelling. This basic principle can be applied to riding aids. According to most teachings on riding, a riding aid consists of two consecutive elements that prompt tension and swelling, and do so in principle as unintended consequences of intentional action. Contraction represents the tension that every riding aid generates when, for example, a stimulus provided by the thigh or by shifting one’s weight to initiate a change in joint movement. Swelling represents the relaxation induced by letting go of the riding aid, by which the rider responds (or should respond) to the horse performing the desired movement. This dual nature of a riding aid as a prerequisite of subtle riding can be found early on in one of the oldest known teachings on riding, Peri Hippikes (On Horsemanship), written by the philosopher Xenophon more than 2000 years ago and which is still referred to today:

“As soon as he raises his neck when you pull, give him the bit at once. Invariably, in fact, as we cannot too often repeat, you must humour your horse whenever he responds to your wishes. And when you notice that high carriage of his neck and lightness of hand give him pleasure, you should not deal hardly with him as though you were forcing him to work, but coax him” [24].

Unlike dancing, for instance, where the partners can also verbally coordinate specific sequences and reach a shared understanding of the meaning of bodily signals, signals between humans and animals can only be communicated nondiscursively, namely, by way of encorporation. Hence, the horse has to find out by trial and error what the riding aid of the rider means, in particular, when it is introduced for the first time to the horse. This communication takes place with no notable temporal differentiation. This is because a riding aid—in the ideal case—always mediates at the same time since it signals a specific movement that the two bodies are to perform in unison, while it also involves feeling, namely, that the quality of the joint performance is felt. It takes effect with a barely perceptible temporal distance, thus both affecting and being affected.

This dual nature of a riding aid is also the reason why horseback riding can take the form of solidary encorporation. Were it to exclusively transmit the will of the rider, it could only take the form of antagonistic encorporation. The interplay between signal and feeling is also reflected in the element of timing mentioned above, namely, that a riding aid should only be applied when the horse is actually able to perform the task. For instance, changing the pace from walking to cantering involves not only recognizing the moment in which the horse lifts its leg to begin to do so but also to sense whether the horse’s whole body is ready to make this transition. For example, does it show the inner “stance”, the necessary tension and “idea”, required to canter.

Horseback riding as embodied communication not only affectively involves both participants but trains them to be attentive to such involvement as well. This mutual training provides the basis for ever-more-refined forms of expression. Bruno Latour considers this training and being trained, which must always be understood in unison, as the foundation of the felt body to begin with. “To have a body is to learn to be affected, meaning ‘effectuated’, moved, put into motion by other entities, humans or non-humans.” ([25], p. 205). In this context, Latour refers to Vinciane Despret, who in turn draws explicitly on human–horse encounters to describe trans-species communication: “Both, human and horse, are cause and effect of each other’s movements. Both induce and are induced, affect and are affected. Both embody each other’s mind.” ([26], p. 115). On the basis of such practices, Despret derives her concept of embodied empathy, which—like Schmitz’s embodied communication—is not limited to human entities and for this reason is also suited for overcoming a mode of thought caught up in human–nature dualisms.

“A concept which describes feeling/seeing/thinking bodies that undo and redo each other, reciprocally though not symmetrically, as partial perspectives that attune themselves to each other. Therefore, empathy is not experiencing with one’s own body what the other experiences, but rather creating the possibilities of an embodied communication” ([27], p. 51).

### 2.2. Solidary Encorporation as Encounter Value

Now, according to Schmitz, solidary embodied communication is strictly speaking only possible if there are at least three parties involved. Two of those are solidary connected with one another, enabled by the third party, who takes the dominant role of the center ([22], p. 41). This center does not have to be a sentient being. Schmitz mentions a piece of music as being a center of a group of people singing together ([22], p. 42). When applied to dancing, this would be the choreography. In the case of horseback riding, this can be a type of movement that is to be performed, for instance, a pirouette or a piaffe, a special riding pattern, or a gait transition.

From the participants’ perspective, solidary embodied communication is not only of an instrumental nature in order to perform a task. My own ethnographic research suggests for riding (as well as for other areas such as dancing) that solidary encorporation is also an end in itself. For millennia, humans have sought to communicate with horses and have continuously concerned themselves with refining this communication—conceptually in the teachings on riding and practically in the everyday practice of riding. People pursue this on instrumental grounds, for instance, when riding horses in war and their life depends on this communication. But, they have also pursued this time and again because they consider the communication with horses as valuable in its own right, specifically the feeling of solidary embodied communication.

Donna Haraway coined the term of trans-species encounter value ([28], p. 46). Encounter value emerges from the trans-species encounter as an end in itself. Being “trans-species” is the crucial element in this context. That is, it does not emerge from the pleasure of solidary encorporation as experienced when dancing but rather by experiencing the world through the body of another being—and feeling the self through that other body.

“It is as if we were moving as one body, but with two heads engaged in an intimate dialogue with one another. Ever more intimate, ever deeper, ever better. But I cannot do that with her [the horse currently being trained] just yet; for now, we are just friends” (Horse trainer, 2017).

Drawing on the example of training young horses, another trainer gives a vivid account of how trans-species encounter value increases over time:

“I pay a lot, really an awful lot of attention to her. Every day. And I’m also with her in the evenings; I talk to her. I also just stand next to her and pat her, put an arm around her, just so that she gets used to it. And also because spending time with her is simply an incredibly beautiful experience (…) Because I really feed off of it. That is, deep down inside, I feed off the fact that the horses like to be with me” (Horse trainer, 2017).

My research and experience lead me to assume that horses, too, seek solidary mutual encorporation. This becomes obvious to every observer when an entire herd of horses runs off simultaneously and synchronous in response to a frightening noise (which is also a suggestion of motion in the Schmitzian sense ([4], p. 10). Also when engaged in daily grazing, horses seem to seek solidary mutual encorporation, for instance, when two horses align themselves perfectly with one another down to minute detail, the posture of their heads and legs and their individual movements mirroring the other, while they show a similar expression, and their private—that is, their individually experienced—atmospheres seem to coalesce. A rider in one of my studies assumes—and most of my interview partners would certainly confirm this—that her horses derive something resembling trans-species encounter value from riding, not always but time to time:

“Whether my horse feels comfortable when we do something together? Even seeks it? Of course, I don’t know. But I do think so—or hope so. There are moments where it is like what I observe among the herd: Biting cold, tension among all of them, they feel the energy in the air. They are all only waiting for a stimulus from somewhere. A rabbit hopping along, a paper bag flying around, a branch cracking. Nothing special—but they all take off. Sometimes I experience something similar when riding. We are both in the right mood; we are both prepared and fully attuned to the other. Then my mare is waiting just as she would be for a plastic bag flying by. But she is waiting for a signal from me. And when I give her a sign, then we have the most beautiful piaffes together. That can also be unconsciously; it’s just that both of us are ready for it” (Rider, 2022).

### 2.3. Riding with Things

What role do nonliving “participants” play in moments of embodied communication between humans and animals? Schmitz repeatedly makes explicit reference to this in his work, for instance, in his example of a cat playing with a ball of wool ([22], p. 24). Considering embodied communication when riding, we can assume that the encorporation of things can be one-sided, as one would expect, but in a specific sense it can also be mutual even though things cannot encorporate anything. Reins or bits in the horse’s mouth (snaffle or bridle) in particular have such a dual nature as they are constitutive of the embodied communication between horse and rider. A trainer describes this vividly in an interview:

“It is much more intimate with a bridle. Think of the bridle as being a phone. Then you feel where the corners of its muzzle are: Whether they are saying, ‘no’, or whether they are ready for a conversation. It tells me so much about the horse’s emotional situation. And perhaps also about what the horse intends to do. And with the bridle, I’m actually inside the horse. And that is such an intimate zone, the mouth of the other. It does sound a bit weird, doesn’t it?” (Horse trainer, 2017).

Things such as bits (but also saddles, spurs, reins, etc.) are key parts of mutual encorporation between horse and rider; they are elements of the collective body that emerge from communication, formed precisely by the horse, the rider, and the mediating objects. In these constellations, riders do not perceive things—as the example demonstrates—as being a one-sided encorporation of a lifeless other but rather as enablers of specific forms of mutual encorporation, also in the solidary mode. At the same time, the one-sided encorporation of things remains relevant, be it by the horse or the human: a saddle that does not fit and inhibits the human rider from moving in rhythm with the horse’s back or bits that do not fit so that the horse is in pain regardless of what the human rider is doing.

Similar to how visual artists increasingly refine their artistic forms of expression over the course of their artistic careers, the embodied communication between horse and rider, in the form of horsemanship, is at best be continuously refined; that is, the two will become more attentive toward one another and sense each other’s subtler nuances. In essence, one can also apply Gugutzer’s (2015) summary conclusion on embodied communication with things in sports to everyday horseback riding, namely, that the antagonistic phases predominate while the solidary phases are mostly the goal aspired to. This particularly happens in situations in which “programs” other than embodied communication as an end in itself take precedence and determine the objective of riding. In these instances, the objects that mediate between rider and horse, such as the bits described above, lose their ability to act as a “telephone” and are more likely to turn into a means of exerting control via pain. A head of a mounted police unit describes this with reference to the example of an “emergency stop”:

“For example, we have police operations where we ride the horse on the curb bit. That means that we have four reins in one hand. Then I have two walkie-talkies, as I am the head of the unit, one device upward to leadership and a device for my officers. And a baton in my other hand. For instance, a large demonstration in Stuttgart, there was very little space and we naturally had to ensure that we were able to work mostly with very precise riding aids, but in some cases also really needed to make a forced stop. When the horse begins to panic. Depending on the horse, using a snaffle may quickly reach its limits [effective intervention may no longer be possible because a snaffle cannot be used to create the amount of pain that curb bits can]” (Head of a mounted police unit, 2019).

The following example furthermore illustrates the need to consider, in analyzing embodied communication, the unequal distribution of the opportunities of communication between the species owing to the given power relationships. On account of this, embodied communication often proceeds violently and can even result in mutilation or death, be it either purely physically, by applying riding aids in ways that cause injury, or with regard to the constitution of the horse overall when encorporation occurs only antagonistically so that the horse can play no active part and thus loses itself in the process:

“A policewoman approached me. She had a onetime police horse. It was no longer fit for this purpose, and she asked me whether I perhaps had an idea what could be done about it. I asked her if I could get a feel for the horse. And then I mounted it. And I had just barely mounted it—I could still cry at the thought—and I was hit by a wave of desperation. I mean, he already looked like a sad horse before. But that, that was a blast of desperation. And then I stood there with the horse, just trying to tell him that I want to be his friend. And after a while, perhaps after 20 min, it seemed that he began to believe me a little bit. And then we started walking. And I had the feeling that, at the end of our conversation, if it were a human being, I could have actually invited him to go to the movies. It was highly emotional. Intimacy to that extent is something that I only experience on a horse, I mean I personally. […] He did not dare convey his feelings. He didn’t even know that he had a say. He just felt like the one who was supposed to function. If he didn’t, it would be ugly for him. That was his experience. Others had the say. I don’t know if that is how they intended it to be or not; I cannot judge that. Whatever the case may be, that horse was ultimately done with” (Horse trainer).

Yet, what the quotation also shows with reference to the rider’s empathy for the maltreated horse is how mutual embodied communication to the utmost degree relies on the ability to show empathy. Conceptually, we can make a connection at this point to Despret’s understanding of empathy, according to which empathy is a prerequisite for embodied communication. Empathy is not merely feeling what the other feels: “it is rather making the body available for the response of another being” ([27], p. 70).

## 3. Sensing Atmospheres in Multispecies Encounters

Atmospheres have similarly great importance for embodied communication. They are always co-communicated when riding and, according to Schmitz, are always tied to situations (on new phenomenology’s concept of atmosphere, see [1]). Riding, feeding, petting, or looking at the landscape together are all examples of “joint trans-species situations” (more on this in the next section). However, the fact that a situation is shared does not necessarily imply that the involved parties experience it in the same way. According to Schmitz, joint situations are frequently intertwined with the “personal situations” of the participants—whether they are humans or animals. This also becomes apparent in the fact that the atmospheres present in situations are subjectively felt differently. This in turn shapes the embodied communication between the participants.

As physical beings, humans and animals fundamentally have in common the ability to experience atmospheres in the form of affective involvement. A rider’s experience with her gelding is a case in point.

“The other day, I had the feeling that my gelding was meditating. He was standing there perfectly still. From afar, I thought he was asleep. But when I came up to him, I saw that he was wide awake. He was looking into the valley, his nostrils wide open, his eyes still, not looking for anything specific, more like taking the valley in as a whole. His body was upright. His ears attentive. As if all of his senses were busy with absorbing the landscape. And then on top of that the horse’s incredible tranquility. My impression was that he was completely within himself and connected with the place. I felt a moment of infinity. The others were grazing” (Rider, 2021).

The quote demonstrates that not only people but also animals seem to sense atmospheres that are, according to Schmitz, visually or climatically related to different times of day, seasons, and landscapes [29]. Their perception, however, is of an utmost subjective nature: whereas one horse is affected by the landscape, the others are busy grazing. In line with Schmitz, this would simply be several atmospheres competing in the same situation. And, it applies to animals as well that if and which atmospheres someone seizes upon depends on the respective state of their felt body as the foundation for their specific resonance with atmospheres, and this state in turn depends on the current state of their personal situation ([30], p. 41).

The fact that horses, too, have a personal situation, a life history, that plays a part in determining how they experience certain moments atmospherically is illustrated by an example of a wild mustang mare that was captured and brought to Germany for profit. Shortly after arriving in Germany, she saw for the first time humans riding horses and horses being ridden by humans. A human sitting on a horse (a member of the herd) in a predatory position appeared to be life-threatening to her and triggered the impulse to run away:

“Then I petted Mausi [name of another horse]. She [the mustang horse] watched that really closely. And then when Maxi rode her. Thus a human on a horse. She just about collapsed when she saw that. She was completely beside herself, completely!” (Horse trainer, 2017).

Riding is a joint trans-species situation in which private atmospheres encounter one another and are (or can be) co-communicated through the interacting bodies. This suggests that the nature of this atmospheric encounter has a substantial influence on the possibilities of solidary embodied communication while riding, for example, if one does not perceive the atmospheric experience of the other and is therefore unable to understand the other’s movements resulting from this.

But, to what extent does the ability exist in principle to experience mutual affective involvement in atmospheres in trans-species encounters? Just as adults cannot immediately experience the emotional state of a child beaming with delight in view of its presents on Christmas because they lack the child’s naïve responsiveness ([30], p. 48), a human can never feel the atmospheres of an animal in the same way that they feel their own, and vice versa. Drawing loosely on von Uexküll [31], we might say that the horse’s world consists of horse things (and the human world of human things), and spatial experiences therefore can ultimately not be shared. Yet, mutual affection is possible nevertheless: in the above example, for instance, the rider was affectively involved in her horse’s meditative experience of the landscape, and this sensitized her to her own experience of the landscape so that—at least as the empirical evidence in this case shows—the “two atmospheres resonate harmoniously”, to put it in Schmitzian terms ([30], p. 48).

Such a capacity for empathy and the bodily feeling of atmospheres can be attributed to horses as well:

“There was a situation. I was outside. And I suddenly had to think of my deceased grandmother, with whom I was really, really close. I was sitting on a block of wood and was extremely sad. Suddenly, my mare was standing next to me. Like above me. Her neck was like an arm protecting me. She didn’t do anything, just showed me, ‘I feel you.’ Or ‘I am with you.’ At least I perceived it that way. That was an incredibly touching situation. She never does that otherwise. She usually stays more to herself” (Rider, 2021).

Along the lines of three dimensions based on Schmitz’s terminology—private/shared, contrastive/harmonious, degree of involvement—different constellations of atmospheres are conceivable, which can be illustrated using the example of human–horse encounters (Table 1). Not least for analytical reasons—namely, the difficulty, even impossibility, of knowing precisely how beings of another kind experience atmospheres—I assume that encounters between private atmospheres are the rule, and “shared atmospheres” are the very rare exception. From this, it follows that the primary issue in trans-species encounters is how private atmospheres interact. Drawing on Schmitz, we can distinguish two levels in this respect: First, are private atmospheres related to each other in terms of being contrastive or harmonious? Second, to what extent is the human or the animal affectively involved in the private atmosphere of the other? Do they not perceive the other’s atmosphere at all? Do they feel it but remain unaffected? Or do they feel the other’s atmosphere and this feeling comes with affective involvement? The conceivable constellations involve different forms of embodied communication (Table 1).

When can we speak of “shared atmospheres” in situations of trans-species encounters? The attempt to observe or directly describe them is not only epistemologically untenable but also problematic from the perspective of the theory of recognition, as this would amount to impermissibly equating the experience of other forms of life with that of humans. I propose to speak of shared atmospheres only when sharing is the prerequisite for mutual solidary encorporation. An argument for something resembling a “shared atmosphere” between human and horse could therefore only be made in light of the result of the interaction but seems indeterminable as such. A case in point is the quotation above in which the rider describes the piaffe situation as one of solidary encorporation based on a shared atmosphere as its constitutive element. The rider and her mare aligned their bodies in a concerted “stance”, which is the prerequisite for the piaffe. Then, “we are simply both ready” (Rider, 2021), and the piaffe happens to them. Rider and horse create the shared atmosphere in an act of mutual encorporation, but once they are “ready”, it happens to both of them equally.

We can apply what Schmitz described for a group of people singing together to this example of horseback riding and more generally to certain other situations of trans-species encounters: that an “atmospheric mood” ([32], p. 35) can emerge, like an air enveloping both human and horse (and a participant observer, as the case may be), one that the two created and befalls them both. The shared atmosphere is the most exhilarating feeling of transcending the boundaries of one’s own life and becoming one with a collective body that can also include the atmosphere of a surrounding landscape.

“There is a certain place near us. The course of a stream on the right, a meadow on the left, where deer are frequently around. You can only go straight ahead, on a grassy path. Ahead is a vast expanse. If the weather is fitting and we are both in a good mood, then we can fly together” (Rider, 2021).

## 4. Horseback Riding as a Joint Situation

In addition to embodied communication and atmosphere, Schmitz’s concept of situations is highly beneficial for human–animal studies—in this context, with an explicit focus on joint trans-species situations—because situations in Schmitz’s understanding allow one to connect the micro phenomena of embodied communication and atmospheric experiences with a macro perspective, that is, overarching human–animal relationships.

According to Schmitz’s new phenomenology, a situation in the sense of new phenomenology is characterized by three features:

“1. It is holistic, i.e., it is set off against the outside and hangs together internally. 2. It hangs together in virtue of a meaningfulness consisting of meaning. Meanings, in the sense intended here, are states of affairs (that something is the case), programs (that something ought to/should be the case [as a norm or as a desire] or problems (whether something is). […] 3. The meaningfulness is internally diffuse in the sense that within it not everything (possibly nothing) is singular […].” ([1], p. 73).

Applied to our example, a typical situation would be a riding course, for instance. Such a course is “holistic” in that it is bound together temporally (from beginning to end) and spatially (indoor riding arena) and is distinguished from other situations of riding by a specific “state of affairs”. A third person is present (the trainer), who provides indications to the horse or the rider or about the interplay between the two and is thus involved in their embodied communication in a manner that guides it. And, there are other people present (other course participants or an audience) who observe the training situation. The situation is “internally diffuse” because, although riding lessons are characterized by a specific subject matter, one can never predict precisely what will define any particular lesson. For embedding embodied communication in the larger context, the “programs” and “problems” present in the situation play a particular role.

*Programs* can be present in a variety of different forms and fashions, even at the same time. They are the given points of reference for action in a situation, for instance, in terms of desires, conventions, or norms. In a riding lesson, for example, a rider can wish to learn an exercise that they previously perceived as being too difficult. The teacher may want to convince the audience that their method of teaching is successful in order to expand their clientele. The horse may be driven by the desire to be with the other members of its herd, which are waiting outside the paddock and are neighing for it to hear. Norms can be implicitly given or codified. An implicit norm in the form of a certain teaching on riding may be hovering over the setting and be guiding rider, teacher, and audience. The rider may want to learn certain lessons out of their specific curriculum in order to acquire a specific certificate that informs about the rider’s training status—or the rider may seek to expand the spectrum of lessons that their horse has learned to improve its training status, as this this may enhance the horse’s value in view of a planned sale. The teacher might explain and correct body images and postures that, according to the doctrine, are considered the ideal-typical mode for horse and rider. The audience may observe both horse and rider critically in terms of whether the teaching and the performance conform with the riding doctrine. In addition, overarching systems of rules are present, such as training in matters of animal welfare, insurance law, and so on; last but not least, a riding arena also has certain codified rules of behavior that everyone is expected to comply with (“stable rules”).

*Problems* can also take on a wide range of different forms and influence the situation. There might be a defective gate to the arena that suddenly creaks in the wind and frightens the horse repeatedly. A sharp headache might absorb all the teacher’s attention. The horse may have a wound where the saddle sits that has been unnoticed and hurts every time the rider applies a certain riding aid, leading to the dysfunction of the particular riding aid and the rider and teacher becoming increasingly exasperated, to the growing amusement of the audience.

These examples indicate the point to be made here: the programs and problems present in situations are always co-communicated in embodied communication between the participants and thus shape how they engage in mutual encorporation. Such programs and problems are not conducive to solidary communication because programs in the form of norms, for instance, cannot be shared as they are not accessible to the horse yet nevertheless shape communication. Disturbances of embodied communication can be situated, they can be single occurrences, and they can remain without lasting impact, but they can also be of a fundamental nature.

For instance, a majority of horses and horse–human relationships and encounters are subject to the agenda of competitive horse riding. Knowledge of this field is necessary if one wants to understand the specific embodied communication between rider and horse in a riding course. For example, competitive horse riding is shaped to a substantial degree by (economic) valorization interests on the part of both the parties directly involved and those in the background such as the breeding industry. Accordingly, embodied communication in this case is not an end in itself but typically tied to the desire to increase the horse’s value. This value can be of a non-monetary nature (e.g., winning trophies, successful track records, audience recognition) and it can be in dollars (e.g., an increase in the sales price of a horse, a higher market price of its offspring, a higher stud fee), which touches upon issues of horses being a “lively commodity” [20,33,34,35].

Subtle riding aids are also welcome in situations such as competitions. But, when the prevailing objectives revolve around money or winning, this also changes the priorities of communication. The German pentathlete Annika Schleu and her national trainer, Kim Raisner, demonstrated this at the 2021 Olympics to an audience of millions when, faced with the prospect of losing the gold medal, they sought to motivate their resistive horse by beating it. In even much more violent form, this practice can be observed every week in the warm-up areas of lower-ranking competitions.

Embodied communication and other micro practices within the human–horse relationship also change as the objectives that define the situation and are co-communicated in these practices change. A look at riding competitions reveals this too. For example, the guidelines for the evaluation of dressage exercises are in constant flux (whereby the interests of the international breeding industry and the power relationships in this context also play a role). This not only systematically promotes giving preference to certain breeds of horses over others (which explains, e.g., why the Lippizaners, once famous for their skills in classical dressage, today are no longer able to win a renowned tournament simply on account of their comparably small size and characteristic gait) but also changes the framework that guides breeding, modes of riding, and training that reaches far beyond the ranks of elite sports. In this way, material-body-centered riding (i.e., riding that focuses on the appearance and movement of the physical bodies of the horse and rider), the objectives of which are reflected in rules and evaluation systems, proliferates in amateur and youth sports. The human–horse encounters in many riding courses—and here we have come full circle—cannot be understood without knowledge of the underlying set of objectives that are largely formed by the interests of powerful (frequently economic) actors, institutions, and discourses in the field of horseback riding.

How economic objectives specifically form situated and embodied communicated trans-species encounters is evidenced by many other examples, for instance, from the leisure industry (horse shows) or fields where horses are utilized as working animals. They have major implications for animal welfare and are often a matter of life and death for the horse. This is because such objectives produce not only images of ideal horse bodies but also of killable life. They create rules for killable life; for example, when horses are no longer fast enough on the race track or their bodies no longer correspond with the ideals of breeding, they find their final economic valorization via the slaughterhouse in the form of animal food.

## 5. Conclusions

Human–animal studies have developed critical positions with regard to the three A concepts that have shaped human–animal relationships for centuries. They are anthropological difference, which is permanently renewed in approaches that build on dichotomies such as nature versus culture (and practically determines and morally legitimizes our dealings with animals); anthropocentrism, which puts human beings at the center of the world in contrast to other forms of life; and anthropomorphism, which projects human experience onto animals’ experience [36,37,38,39]. The task is to develop theoretical positions and methods to address these criticisms and deal with the three A concepts productively [40].

Schmitz’s new phenomenology can provide a fruitful path toward achieving this. This applies in particular to his conceptualization of embodied communication, and it does so in two ways. On the one hand, it does so empirically: as an analytical concept, embodied communication points the way toward the multiply contoured natural–cultural contact zones that span the range of human–animal encounters and shows how the seemingly clear boundaries between the species become fluid and humans and horses—or humans and dogs, and so on—mutually constitute each other in such communication. And, on the other hand, it does so epistemologically: embodied communication is not just an analytical concept or an object of research but also a mode of knowledge production. This is because scientists, too, communicate via their bodies with their research objects. Accepting embodied communication as a source of knowledge can thus contribute to looking differently at the world and being more attentive, theoretically and methodologically, to nonhuman forms of life. The fact that the concept of embodied communication provides empirical access to how humans communicate with, and, in so doing, become attentive to, other life forms also sensitizes them to other forms of attention [41].

The concept can thus be a building block for human–animal studies to move beyond attempts to confirm anthropological differences in an essentializing manner and focus on the importance of embodied communication in the everyday practice of shared lifeworlds and ask how humans draw—or also overcome—boundaries in their encounters with animals in order to question the conditions for the emergence of these boundaries. As embodied communication is conceived as postdualist in principle, the concept is also suited to overcome the anthropocentric view—or at least to sensitize us to the fact that epistemological anthropocentrism, although unsurmountable, can be critically reflected upon. To delve deeper into the question of how embodied communication conceptualizes our understanding of processes of (scientific) knowledge production and can make a valuable contribution, it would be promising to think more in-depth about linking approaches from the sociology and philosophy of knowledge with approaches from new phenomenology.

The concept of embodied communication would seem to tie in with other (social) theories. For it to enrich empirical analyses would require incorporating methods that move the felt body as a combining element to the center of attention and, in so doing, also attempt to give greater voice to nonhuman forms of life [42,43,44,45]. Combining the concept of embodied communication with new phenomenology’s concept of situation furthermore enables research to start at the micro level of concrete encounters between humans and animals, which are always characterized by such communication, while going beyond to obtain a grasp of fundamental questions, such as how society organizes its relationship with animals.

## Figures and Tables

**Table 1:** Atmospheric constellations in trans-species encounters as a “joint situation” (the example of humans and horses).

Extent of Mutual Affectedness	Private Atmospheres		Shared Atmosphere
	Contrastive, Not Mutually Related	Harmonious, Mutually Related	Part of Solidary Encorporation
**Not perceived**	A rider affected by the spectacle of sunrise fails to notice panic overcoming the horse in response to an approaching herd of wild boars. The horse bolts; the rider falls off.	X	X
**Sensed**	A relaxed horse senses a human’s distractedness and hecticness because of an upcoming business meeting. It turns away and leaves.	An owner senses the delight that the view of a green pasture in springtime triggers in her gelding. He grazes and she enjoys the quiet.	X
**Affectively involved**	A horse shows anxiety on an obstacle course and does not want to subject itself to it; the rider fears the loss of a medal and attempts to take the horse through the course by force. A fight begins.	A mare experiences her owner feeling sad. She bends over her to convey “I feel you”, “I am with you”.	A mare and her rider share a bodily “stance” for executing a piaffe, which then “happens”.

## Data Availability

The original contributions presented in the study are included in the article, further inquiries can be directed to the corresponding author.

## References

[1] Schmitz H. (2019). New Phenomenology. A Brief Introduction.

[2] Uzarewicz M. (2011). Der Leib und die Grenzen der Gesellschaft: Eine Neophänomenologische Soziologie des Transhumanen.

[3] Gugutzer R. (2020). Beyond Husserl and Schütz. Hermann Schmitz and Neophenomenological Sociology. J. Theory Soc. Behav..

[4] Schmitz H. (2017). The Felt Body and Embodied Communication. Yearb. East. West. Philos..

[5] Birke L., Hockenhull J., Birke L., Hockenhull J. (2012). On Investigating Human-Animal Bonds: Realities, Relatings, Research. Crossing Boundaries: Investigating Human-Animal Relationships.

[6] Pütz R., Hasse J., Schreiber V. (2019). Pferderücken. Räume der Kindheit: Ein Glossar.

[7] Pütz R., Borgards R., Felcht F., Kuni V., Middelhoff F., Pütz R., Schlottmann A. (2024). Sporen. Von Fliegenfängern und Katzenklappen. 39 Kleinigkeiten Zwischen den Arten.

[8] Maurstad A., Davis D., Cowles S. (2013). Co-being and intra-action in horse-human relationships: A multi-species ethnography of be(com)ing human and be(com)ing horse: A multi-species ethnography of be(com)ing human and be(com)ing horse. Soc. Anthropol. Anthropol. Soc..

[9] Birke L., Brandt K. (2009). Mutual corporeality: Gender and human/horse relationships. Women’s Stud. Int. Forum.

[10] Schuurman N. (2021). Animal work, memory, and interspecies care: Police horses in multispecies urban imaginaries. Cult. Geogr..

[11] Coulter K. (2016). Animals, Work, and the Promise of Interspecies Solidarity.

[12] Hagis E., Gillespie J. (2021). Kosciuszko National Park, Brumbies, law and ecological justice. Aust. Geogr..

[13] Pütz R., Schlottmann A. (2020). Contested conservation—Neglected corporeality: The case of the Namib wild horses. Geogr. Helv..

[14] Pütz R. (2017). Wildpferde in den USA: Ressourcenkonflikte, Wildniskonstruktionen und Mensch-Wildtier-Verhältnisse. Geogr. Rundsch..

[15] Rikoon J. (2006). Sanford. Wild horses and the political ecology of nature restoration in the Missouri Ozarks. Geoforum.

[16] Merkies K., Franzin O. (2021). Enhanced Understanding of Horse-Human Interactions to Optimize Welfare. Animals.

[17] Kelly K.J., McDuffee L.A., Mears K. (2021). The Effect of Human-Horse Interactions on Equine Behaviour, Physiology, and Welfare: A Scoping Review. Animals.

[18] Lesimple C. (2020). Indicators of Horse Welfare: State-of-the-Art. Animals.

[19] Kornherr E., Pütz R. (2022). Othering, governing, and resistance of abject urban animals: Egyptian geese and their right to the city. Political Geogr..

[20] Pütz R. (2021). Making companions: Companionability and encounter value in the marketization of the American Mustang. Environ. Plan. E Nat. Space.

[21] Pütz R., Poerting J. (2020). Mensch-Tier-Verhältnisse in der Konsumgesellschaft. Berichte. Geogr. Landeskd..

[22] Schmitz H. (1998). System der Philosophie. Fünfter Band: Die Aufhebung der Gegenwart, 2. Auflage.

[23] Hunt R. (1978). Think Harmony with Horses. An in-Depth Study of Horse/Man Relationship.

[24] Xenophon (1925). On Horsemanship.

[25] Latour B. (2004). How to talk about the body? The normative dimension of science studies. Body Soc..

[26] Despret V. (2004). The Body We Care for: Figures of Anthropo-zoo-genesis. Body Soc..

[27] Despret V. (2013). Responding Bodies and Partial Affinities in Human–Animal Worlds. Theory Cult. Soc..

[28] Haraway D. (2008). When Species Meet.

[29] Schmitz H. (2016). Atmospheric Spaces. Ambiances.

[30] Schmitz H., Fink-Eitel H., Lohmann G. (1993). Gefühle als Atmosphären und das affektive Betroffensein von ihnen. Zur Philosophie der Gefühle.

[31] Uexküll J.J. (2010). von. A Foray into the Worlds of Animals and Humans: With a Theory of Meaning.

[32] Schmitz H. (1998). Der Leib, der Raum und die Gefühle.

[33] Gillespie K. (2021). The afterlives of the lively commodity: Life-worlds, death-worlds, rotting-worlds. Environ. Plan. A.

[34] Collard R.-C., Dempsey J. (2013). Life for sale? The politics of lively commodities. Environ. Plan. A Econ. Space.

[35] Collard R.-C. (2020). Animal Traffic.

[36] Urbanik J. (2012). Placing Animals: An Introduction to the Geography of Human-Animal Relations.

[37] Smith J.A., Mitchell R.W., Smith J.A., Mitchell R.W. (2012). Animal Ethics and Animals’ Minds: Reflections. Experiencing Animal Minds: An Anthology of Animal-Human Encounters.

[38] Philo C., Wilbert C. (2000). Animal Spaces, Beastly Places: New Geographies of Human-Animal Relations.

[39] Lorimer J. (2015). Wildlife in the Anthropocene: Conservation after Nature.

[40] Borgards R., Jaeger F. (2020). Cultural Animal Studies zwischen neuer Tiertheorie und New Ethology. Menschen und Tiere. Grundlagen und Herausforderungen der Human-Animal Studies.

[41] Despret V. (2022). Living as a Bird.

[42] Kirksey S.E., Helmreich S. (2010). The emergence of multispecies ethnography. Cult. Anthropol..

[43] Gillespie K. (2021). For multispecies autoethnography. Environ. Plan. E Nat. Space.

[44] Colombino A., Bruckner H.K. (2023). Methods in Human-Animal Studies: Engaging with Animals Through the Social Sciences.

[45] Schröder V., Steiner C., Rainer G., Schröder V., Zirkl F. (2022). Tierliche Lebenswelten verstehen lernen?: Perspektiven mehr-als-menschlicher Ethnographien. Mehr-als-Menschliche Geographien: Schlüsselkonzepte, Beziehungen und Methodiken.

